# Enhancement of zaleplon oral bioavailability using optimized self-nano emulsifying drug delivery systems and its effect on sleep quality among a sample of psychiatric patients

**DOI:** 10.1080/10717544.2019.1687613

**Published:** 2019-11-21

**Authors:** Maha K. A. Khalifa, Hoda A. Salem, Seham M. Shawky, Heba A. Eassa, Asmaa M. Elaidy

**Affiliations:** a Department of Pharmaceutics and industrial Pharmacy, Faculty of Pharmacy, Al-Azhar University, Nasr City, Egypt;; b Department of Clinical Pharmacy, Faculty of Pharmacy, Al-Azhar University, Nasr City, Egypt;; c Department of Psychiatry, Faculty of Medicine for girls, Al-Azhar University, Nasr City, Egypt

**Keywords:** Zaleplon, self-nano emulsifying drug delivery system (SNEDDS), bioavailability, poorly water-soluble drug and quality of sleep

## Abstract

The aim of this work is to develop self-nano emulsifying drug delivery system (SNEDDS) to enhance the oral bioavailability of zaleplon (Zal) as a poorly water-soluble drug. Moreover, the bioavailability and the effect on the quality of sleep among a sample of psychiatric patients is to be assessed. D-optimal mixture design was used for optimization. Optimized SNEDDS formulation was evaluated for droplet size, transmission electron microscope (TEM) and in-vitro dissolution test. Zal bioavailability was evaluated by determining its serum concentration and pharmacokinetic parameters in 8 patients after oral administration. Effect on sleep quality was assessed among 40 psychiatric patients. Patients’ sleep quality was assessed in 40 psychiatric patients before and after medication using the Arabic version of the Pittsburgh Sleep Quality Index (PSQI). Zal- SNEDDS appeared as nano-sized spherical vesicles. Moreover, Zal was completely dissolved from optimized formulation after 45 min indicating improved dissolution rate. Zal-SNEDDS showed significantly higher *C*_max_, *T*_max_ and *AUC*_0→∞_ compared to commercial product after oral administration. Zal-SNEDDS significantly improved the total score of PSQIs (*p* < .001) with higher subjective sleep quality, reduced sleep latency, improved day time function and sleep disturbance (*p* < .001). Using sleep medication was reduced significantly (*p* = .027). However, it did not modify sleep duration or sleep efficiency. SNEDDS have improved Zal solubility and enhanced its bioavailability. Furthermore, Zal-SNEDDS have improved the total score of PSQIs and may be considered a good choice to enhance the quality of sleep among psychiatric patients.

## Introduction

Sleep problems are increased among psychiatric patients with marked and complex bi-directional causality; the diagnostic criteria for many psychiatric conditions include sleep problems, as well as some sleep disorders, may increase the risks of developing episodes of psychiatric disorders (Krystal, 2012). Zaleplon (Zal) is a pyrazolopyrimidine hypnotic drug, prescribed for the short-term management of insomnia. Additionally, it is a potential anticonvulsant which minimizes the convulsions induced by phenylenetetrazole and electroshock by acting on the GABA receptor (Jablan et al., 2011; Janga et al., 2013). Zal is categorized as Class II drugs, according to the bio-pharmaceutical classification system (BCS) (Teixeira et al., 2017; Abd-Elrasheed et al., 2018), showing poor solubility and high permeability which explain its poor dissolution rate, delayed onset of action and limited absorption (Dudhipala, 2016). Moreover, Zal bioavailability is about 30% as a result of extensive first-pass metabolism (Drover, 2004). Hallucination is one of the main drawbacks of Zal due to its high dose (Gunja, 2013; Narendar et al., 2016). Different strategies have been adopted by formulators to improve the bioavailability of Zaleplon. Solid dispersions, oro-dispersible films, inclusion complexes using cyclodextrin and micro-/nanoemulsions are examples for these strategies (Waghmare et al., 2008; Abd-Elrasheed et al., 2018; Manda et al., 2018). Nano-scale technologies/carriers have been utilized for improving solubility of hydrophobic drugs and hence their therapeutic performance. The self-nano emulsifying drug delivery system (SNEDDS) is one of the most practical systems to overcome low solubility and poor oral absorption of water-insoluble drugs (Shahba et al., 2012). SNEDDS are anhydrous isotropic mixtures of oil, surfactant(s) and drug which, when introduced into the aqueous phase (GIT) with gentle agitation, spontaneously form O/W nanoemulsions (with globule size less than 200 nm) (Xi et al., 2009). O/W nanoemulsion provides interfacial area for drug portioning between oil and GI fluid (Singh & Pai, 2015). Therefore, SNEDDS provides efficient strategy to enhance lipophilic drug oral bioavailability (Jeevana & Sreelakshmi, 2011). Furthermore, SNEDDS allow drug to bypass hepatic first-pass metabolism and overcome intestinal metabolism conducted by Cytochrome P450 isoforms 7 (Singh et al., 2011). The objective of this study is to minimize Zaleplon drawbacks by improving Zal solubility and enhancing its bioavailability through SNEDDS. Furthermore, Zal-SNEDDS effect on sleep quality in psychiatric patients will be assessed.

## Material and methods

### Material

Zaleplon was kindly donated as a gift from Al-Andals Pharmaceuticals Company (Egypt), labrafil & (Propyelene glycol mono caprylate) capryol, Labrosol and transcutol were obtained from Sigma Aldrich (St. Louis, USA). Ethanol was purchased from ADWIC, (Egypt). All other materials were of analytical grade.

### Methods

#### Preparation of SNEDDS

A series of SNEDDS was prepared with two types of oil (labrafil & capryol). Zal was dissolved in the surfactant in glass vials. Oil and co-surfactant were accurately weighed into glass vials. Then, the components were mixed by vortex mixing, and heated at 37 °C in an incubator to obtain a homogeneous isotropic mixture. The SNEDDS formulations were stored at room temperature until used.

#### Optimization of Zal-SNEDDS formulations using d-optimal mixture design

**Experimental design.** In order to reduce the trial numbers of the experiments needed for the optimization of Zal-SNEDDS formulations, a response surface randomized l optimal (Design-Expert R software version 10 stat-ease Inc., Minneapolis, MN, USA) was used. In this design, the effect of oil percent, surfactant: co-surfactant ratio and oil type on the particle size and optical clarity of Zal-SNEDDS was studied. The experimental range for independent variables and responses are shown in Table 1.

**Table 1.** The experimental range for independent variables and responses.

Factors	Levels		
	−1	0	1
X_1_: Oil percent	10	15	20
X_2_: surfactant: co-surfactant	1:1	1.5	2
X_3_: Oil type	Capryol	Labrafil	
Response	Constraints		
Y_1_: optical clarity (light absorbance by diluted SNEDDS)	Minimize		
Y_2_: Particle size (mean droplet size)	Minimize		

### Characterization of SNEDDS

#### Spectroscopic characterization of optical clarity

The absorbance as a measurement of optical clarity for the prepared SNEDDS formulations was measured spectrophotometrically using Hitachi UV-Vis spectrophotometer (Hitachi, Japan) after adequate dilution with distilled water. The SNEDDS formulations equivalent to 5 mg Zal were diluted with distilled water (1:100) and analyzed at 400 nm using distilled water as the standard blank solution (Fahmy et al., 2015; Nasr et al., 2016a; 2016b).

#### Particle size measurement

Particle size of the prepared SNEDDS formulations was measured using a Zeta-sizer 3000 PCS (Malvern Instr., England) equipped with a 5mW helium-neon laser with a wavelength output of 633 nm. Measurements were made at 25 °C, angle 90°, run time at least 180 sec. Data interpreted by the method of cumulants. The samples were diluted with double-distilled water (1:100) prior to the measurements. The particle size values given are the averages of 3 measurements over 5 min each (Yadav et al., 2014; Parmar et al., 2015).

#### Transmission electron microscopy (TEM)

The optimized SNEDDS formulation (F12) was diluted with water (1:1000), a drop of the sample was placed onto a mesh carbon-coated copper grid and allowing the sample to settle for 35 min. Excess fluid was then removed by wicking it off with an absorbent paper., then stained with uranyl acetate stain and examined by transmission electron microscope (JEM 1010, JEOL Ltd, Tokyo, Japan) with an acceleration voltage of 70 kV and magnification power of 150 KX 19.

#### Encapsulation of Zal- SNEDDS

Weighed amount of optimized SNEDDS (F12) equivalent to 5 mg was filled in hard gelatin capsules size ‘00’. Filled capsules (CF12) were stored at room temperature for 24 hrs before their use in subsequent studies. Zaleplon powder was filled in the same capsule size and used as a control for comparison.

#### *In-vitro* dissolution studies

Dissolution of CF12 was studied compared to control Zal capsules using USP dissolution apparatus type II (paddles) rotating at 75 rpm (Manda et al., 2018). The dissolution was performed in 300 ml of HCl (pH1.2) at 37 ± 0.5 °C. Samples were withdrawn at 1, 2, 3, 4, 5, 10, 15, 30, 45, 60, 90 and 120 min and replaced by equal volumes of fresh release medium maintained at the same temperature. Samples were filtered by passing through 0.45 mm membrane filter (Millipore, Billerica, MA, USA), and spectrophotometrically assayed for drug content at 232 nm (Jablan et al., 2011; Jeevana & Sreelakshmi, 2011). Dissolution efficiency was calculated for CF12 compared to control Zal capsule using the following equation:

$$DE=\frac{\int_{t_{1}}^{t_{2}}y\cdot dt}{y_{100}\times (t_{2}-t_{1})}\times 100$$

where, *y* is the percentage of dissolved product, DE is the area under the dissolution curve between two time points (*t*_1_ and *t*_2_) and represent the percentage of the curve at maximum dissolution, *y*_100_, over the same time period (Kassaye & Genete, 2013).

## Determination of clinical pharmacokinetic parameters after oral administration of Zal-SNEDDS

Sixteen psychiatric patients aged 33.56 ± 2.77 years (mean ± standard deviation) participated in the study. Patients were being randomized into two groups; Group I: Eight psychiatric patients administered a single oral dose of 5 mg Zal- SNEDDS and Group II: Eight psychiatric patients administered a single oral dose of Zal commercial product; (Sleep-aid)^®^ October Pharm Company 5 mg tablet. A control blood sample was taken just before drug administration. Blood samples were drawn after 0.5, 1, 2, and 4 hr. Blood samples were immediately centrifuged at 300 rpm for 10 min, and the serum was separated and frozen at −20° C until assayed for Zal concentration. Zal was quantitatively determined in serum by high performance liquid chromatography (HPLC). 200 μL of serum sample was added to 50 μL of trichloroacetic acid, vortexed for 10 sec. The supernatant was filtered through filter paper 0.45 mm and directly injected into the HPLC system (LC1620A Liquid Chromatograph, made in Korea). The detector was UV and absorbance was at 338 nm. Injected volume was 20 μL at a flow rate of 1 ml/min. Elution buffer was acetonitrile with 5% Acetic acid, Column: C18 with manual injection (Metwally et al., 2007). The data obtained were subjected to unpaired “*t*” student test with Instat Graphpad prism software (version 4.00; GraphPad Software, San Diego, CA, USA). The level of statistical significance was chosen as less than *p* < .05.

## Sleep quality assessment among psychiatric patients treated with Zal-SNEDDS

### Patients and methods

#### Study design

A cross-sectional comparative case-control study was conducted at Al-Zahraa University Hospital, Psychiatry Department affiliated to Faculty of Medicine for Girls, Al-Azhar University, Egypt. The patients were identified by coded numbers to maintain privacy. After approval of the Medical Ethics Committee number (RHDIRB 2018122001) and written informed consent from the patients, the study was carried out. Inclusion criteria were female and male psychiatric patients complained of insomnia and aged between 22 and 60 years. The patients who did not fulfill the previous inclusion criteria, patients with delirium, mental retardation or language problems, patient with acute or chronic physical diseases, pregnancy or breastfeeding, history of allergy to zaleplon or reported history of substance abuse were excluded from the study. A total of 40 psychiatric patients suffered insomnia were divided into two equal groups; the first group was given CF12 (Zal-SNEDDS formula 5 mg)/day at 10 pm for 7 days and the second group was given (Sleep-aid)^®^ 5 mg tablet/day for 7 days. Patients of both groups were blinded to the drug administered. Patients’ adherence to the treatment was checked by the psychiatrists via checking the number of medications left and their blisters. Sleep quality of each group was assessed before treatment using the Arabic version of the Pittsburgh Sleep Quality Index (PSQI) (Buysse et al., 1989). All the PSQI forms were filled out by a professional psychiatrist. The PSQI is nine questions, 19 items self-report instrument designed to measure sleep quality and disturbance over a one-week period. The PSQI measures seven components; 1- subjective sleep quality, 2- sleep latency (amount of time that it takes to fall asleep), 3- sleep duration, 4- habitual sleep efficiency (hours between bedtime and waking up time), 5- sleep disturbances, 6- use of sleeping medications, and 7- daytime dysfunction over the previous month. Each of these areas was self-rated by the patient him/herself. Answers were scored on an ordinal scale from zero to three where three reflects the lowest quality. The sleep quality index score was calculated as the sum of the seven components score where scores over five indicate clinically disturbed or poor sleep quality. Sleep quality was re-assessed after a one week of medication.

#### Statistics

PSQI forms were extracted and coded in Microsoft Excel 2016 and prepared for further data analysis. Data for (Sleep-aid)^®^ and Zal-SNEDDS groups were separated for the purpose of data comparison. Patient’s qualitative data of each group as sex, marital status, education, occupation, mental illness, and medication including levels of each variable were subjected to frequency analysis. Descriptive statistics including mean, median, standard error of mean, minimum, maximum, range, and quartiles were calculated for age as the only quantitative variable and for the sleep quality aspects and the total index before and after data as ordinal variables. Both descriptive statistics and frequency analysis were done using IBM SPSS ver. 23.

Sleep quality data of the two groups were compared. In order to investigate the effect of each medication on the sleep quality within each group, Patient’s data before and after treatment within each group were compared. Data of the two groups of patients before treatment were compared to assure the resemblance between the two groups at the seven aspects of sleep quality and consequently eliminate the effect of variation between the two groups on the final results after treatment. The after-treatment data of the two groups were also compared to investigate the efficacy of both treatments against each other.

All comparisons were carried out using SigmaPlot^®^ 12.5. Assumptions of normality were assessed using Shapiro–Wilk’s test and results showed significant results (*p* < .05). Failing to meet the assumptions of the parametric t-tests has led to the use of the non-parametric tests for all the seven aspects of sleep quality and for the total index as well. Wilcoxon Signed Rank Test was used for the paired data of the before- and after-treatment comparisons within each group while Mann-Whitney rank-sum test was used to compare the independent data between the two groups (before vs. before and after vs. after). Data of all comparisons were plotted using Boxplot graphs in order to represent all aspects of non-parametric data distribution. Data visualizations were carried out using Minitab ver. 18.1.

## Results and discussion

### Response surface randomized l optimal design and analysis

Fourteen experiments were carried out according to the experimental design. Composition and the observed responses of the design are illustrated in Table 2.

**Table 2.** Composition and the observed responses of the design.

Run	X_1_	X_2_	X_3_	Y_1_	Y_2_
1	1	1	Labrafil	0.525 ± 0.02	88.9 ± 1.01
2	0	0	Labrafil	0.484 ± 0.01	80 ± 1.07
3	−1	0	Capryol	0.787 ± 0.04	122.67 ± 0.94
4	1	−1	Capryol	0.592 ± 0.05	120.27 ± 0.85
5	1	0	Capryol	0.454 ± 0.01	97.3 ± 0.72
6	0	−1	Labrafil	0.380 ± 0.02	68 ± 0.94
7	−1	1	Labrafil	0.698 ± 0.04	113.07 ± 1.2
8	0	0	Capryol	0.636 ± 0.01	111.98 ± 1.65
9	0	−1	Capryol	0.761 ± 0.05	134.9 ± 1.19
10	−1	1	Capryol	0.653 ± 0.02	101.7 ± 1.07
11	1	−1	Labrafil	0.312 ± 0.06	56.42 ± 0.64
12	−1	−1	Labrafil	0.431 ± 0.08	79.79 ± 2.23
13	1	1	Capryol	0.261 ± 0.01	74.3 ± 1.46
14	0	1	Capryol	0.458 ± 0.05	87.01 ± 2.59

### Effect of independent variables on optical clarity

From the model summary statistics of models, it was concluded that the statistically significant model detected was 2FI model. Table 3 shows the ANOVA study of different factors on optical clarity.

**Table 3.** Analysis of variance for 2FI model for different factors on optical clarity.

Source	Sum of squares	Df	Mean square	F value	*p*-value Prob > F
Model	0.34	6	0.057	343.76	<.0001
X_1_	0.13	1	0.13	784.87	<.0001
X_2_	2.58E-003	1	2.580E-003	15.56	.0056
X_3_	0.049	1	0.049	293.86	<.0001
X_1_ X_2_	1.122E-003	1	1.122E-003	6.77	.0354
X_1_ X_3_	0.023	1	0.023	136.18	<.0001
X_2_ X_3_	0.17	1	0.17	1004.99	<.0001
Residual	1.161E-003	7	1.658E-003		
Cor Total	0.34	13			

The model f value of 343.78 implies the model is significant. There is only a 0.01% chance that an F-value this large could occur due to noise. Values of Prob > F less than 0.0500 indicates model terms are significant. In this case x_1_, x_2_, x_3_, x_1_x_2_, x_1_x_3_ and x_2_x_3_ are significant model terms. It was found that the predicted R-Squared of 0.9902 is in reasonable agreement with the adjusted R-Squared of 0.9937. Adequate Precision measures the signal to noise ratio. A ratio greater than 4 is desirable. In this case, the ratio of 57.092 indicates an adequate signal and this model can be used to navigate the design space.

Final equation in terms of coded factors was derived as

$$\text{Optical}\;\text{clarity}\left(Y_{1}\right)=0.55-0.13X_{1}-0.017X_{2}-0.062X_{3}-0.013X_{1}X_{2}+0.052X_{1}X_{3}+0.14X_{2}X_{3}$$

The equation in terms of coded factors can be used to make predictions about the response for given levels of each factor. Optical clarity indicated by light absorbance of diluted SNEDDS was studied. In case of Capryol: Y_1_ = 0.61471 − 0.17753 X_1_ − 0.15190 X_2_ − 0.013452 X_1_ X_2_.

From equation, it was found that maximum clarity indicated by lowest light absorbance can be achieved by increasing oil% and Surfactant: cosurfactant ratio. While in case of labrafil, maximum clarity can be achieved by increasing oil% and decreasing Surfactant: cosurfactant ratio as presented in the equation: Y_1_ = 0.49138 − 0.073X_1_ + 0.11828 X_2_ − 0.013452 X_1_ X_2_.

**Figure 1.**
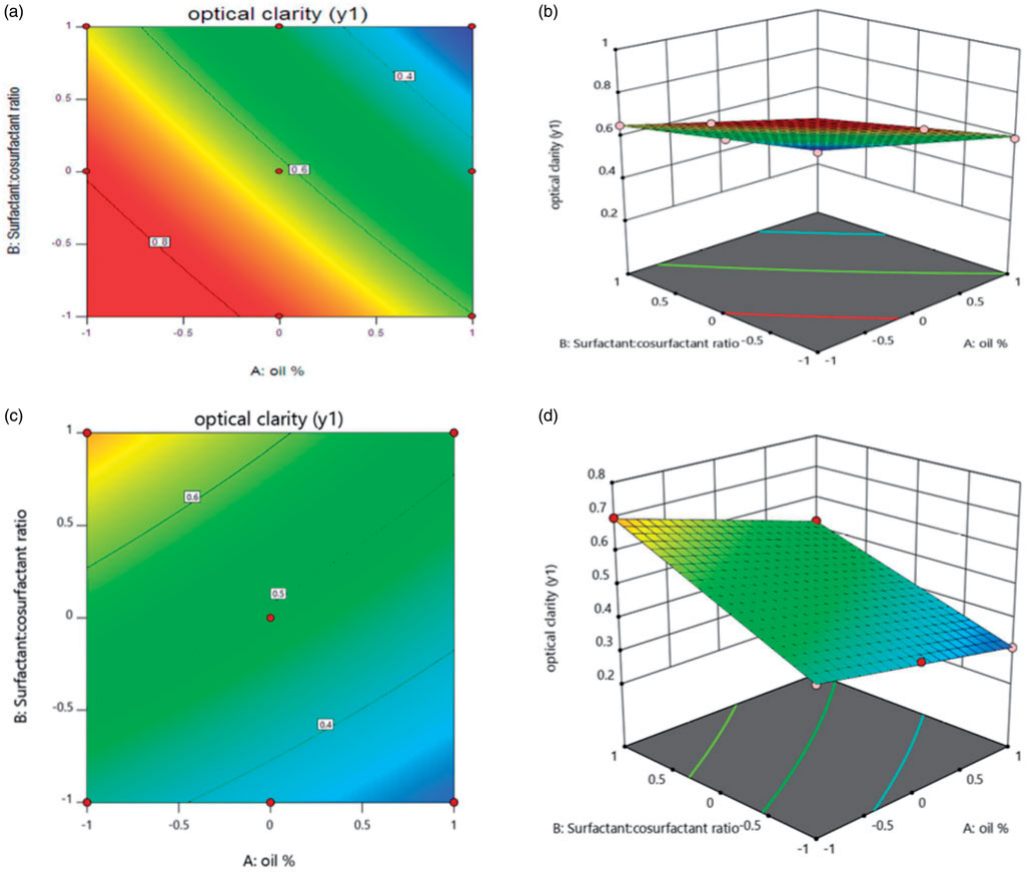
The effect of factorial variable on optical clarity in case of Capryol (a: Contour plot, b: 3 D response-surface) and labrafil (c: Contour plot, d: 3 D response-surface).

The relationship between the dependent and independent variables were further elucidated using contour plots and response surface plot (Figure 1).

### Effect of independent variables on particle size

From the model summary statistics of models, it was concluded that the statistically significant model detected was 2FI model. Table 4 shows the ANOVA study of different factors on particle size.

**Table 4.** Analysis of variance for 2FI model for different factors on particle size.

Source	Sum of squares	df	Mean square	F value	*p*-value Prob > F
Model	6839.30	6	1139.88	356.38	<.0001
X_1_	1315.4	1	1315.04	411.14	<.0001
X_2_	109.06	1	109.06	34.10	.0006
X_3_	2345.21	1	2345.21	733.22	<.0001
X_1_ X_2_	0.18	1	0.18	0.055	.8210
X_1_ X_3_	3.99	1	3.99	1.25	.3011
X_2_ X_3_	3564.23	1	3564.23	1114.34	<.0001
Residual	22.39	7	3.20		
Cor Total	6861.69	13			

The model f value of 356.38 implies the model is significant. There is only a 0.01% chance that an F-value this large could occur due to noise. Values of Prob > F less than 0.0500 indicates model terms are significant. In this case x_1_, x_2_, x_3_ and x_2_x_3_ are significant model terms. It was seen that the predicted R-Squared of 0.9799 is in reasonable agreement with the adjusted R-Squared of 0.9939. Adequate Precision measures the signal to noise ratio. A ratio greater than 4 is desirable. In this case, ratio of 61.908 indicates an adequate signal and this model can be used to navigate the design space.

Final equation in terms of coded factors was derived as

$$\text{Particle}\;\text{size}\left(Y_{2}\right)=97.28-12.58X_{1}-3.46X_{2}-13.53X_{3}-0.17X_{1}X_{2}+0.69X_{1}X_{3}+19.76X_{2}X_{3}$$

Final equation in terms of actual factors:

$$\text{In}\;\text{case}\;\text{of}\;\text{Capryol}:Y_{2}=110.80559-13.27005X_{1}-23.21339X_{2}-0.16871X_{1}X_{2}$$

From equation, it is clear that the particle size is decreased by increasing the oil % as well as increasing surfactant: cosurfactant ratio. While in case of labrafil, the particle size is decreased by increasing the oil % and decreasing surfactant: cosurfactant ratio as illustrated in the following equation: Y_2_ = 83.74690 − 11.88500 X_1_ + 16.30138 X_2_ − 0.16871 X_1_ X_2_.

**Figure 2.**
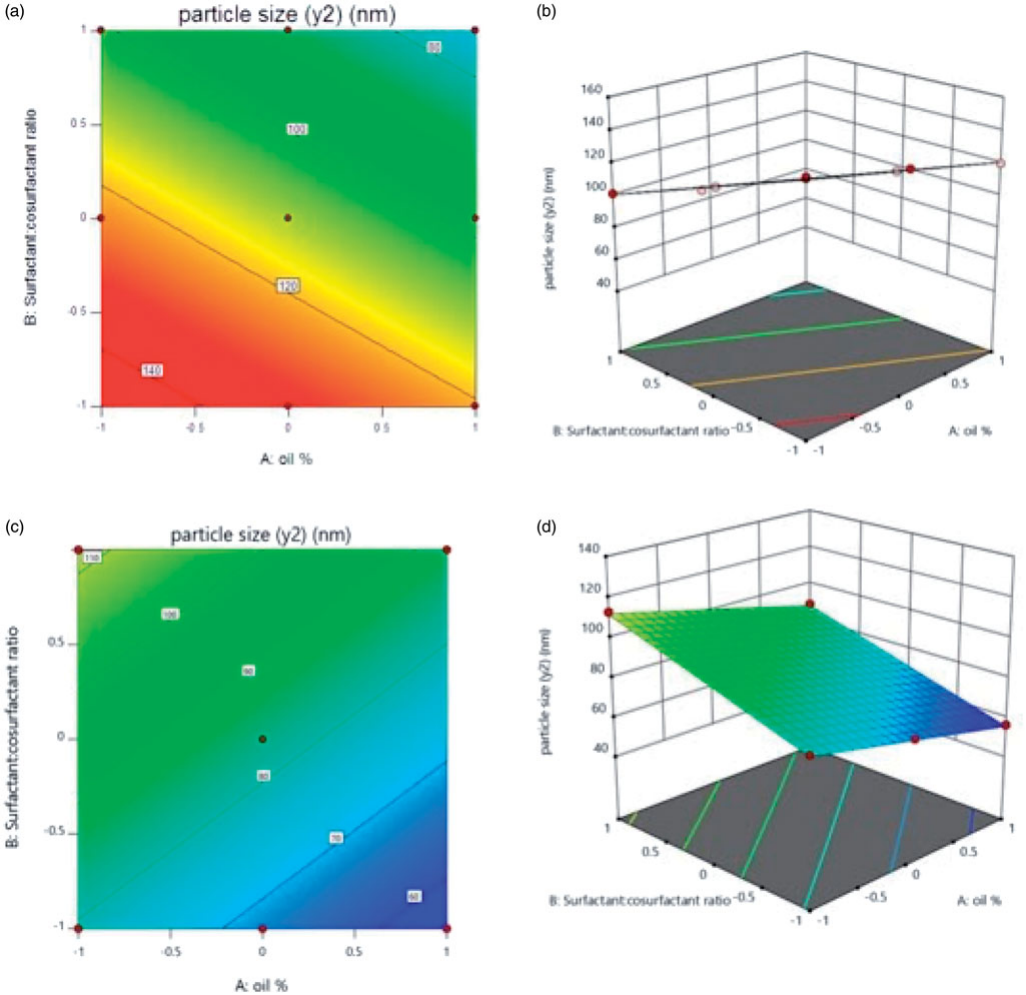
The effect of factorial variable on particle size in case of Capryol (a: Contour plot, b: 3 D response-surface) and labrafil (c: Contour plot, d: 3 D response-surface).

The relationship between the particle size and independent variables were further elucidated using contour plots and response surface plot (Figure 2).

#### Optimization

**Figure 3.**
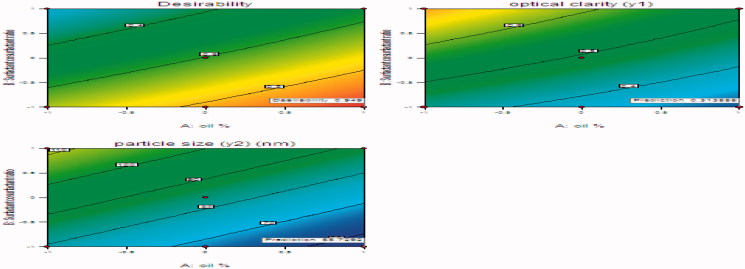
Contour and response surface methodology desirability plot for optimum results.

after generating the polynomial equations concerning the dependent and independent variables, the optimum formulation was chosen based on the conditions for attaining the maximum optical clarity indicated by minimum absorbance and minimum particle size. The optimization procedure was conducted automatically by the design expert and based on utilizing the desirability function. Figure 3 represents an overlay plot showing the optimized formulation chosen by the software to obtain the required responses. The optimized formulation was achieved with 20% Labrafil and 1:1 surfactant: co-surfactant. The predicted optical clarity for optimized formulation is 0.31355 and its observed optical clarity is 0.303. The predicted and observed particle size for optimized formulation are 55.7292 and 51.44, respectively. There is a small residual between the predicted and observed responses, so these results demonstrate the validity and reliability of the optimization procedure used in prediction of the formulation variables.

### Transmission electron microscopy (TEM)

**Figure 4.**
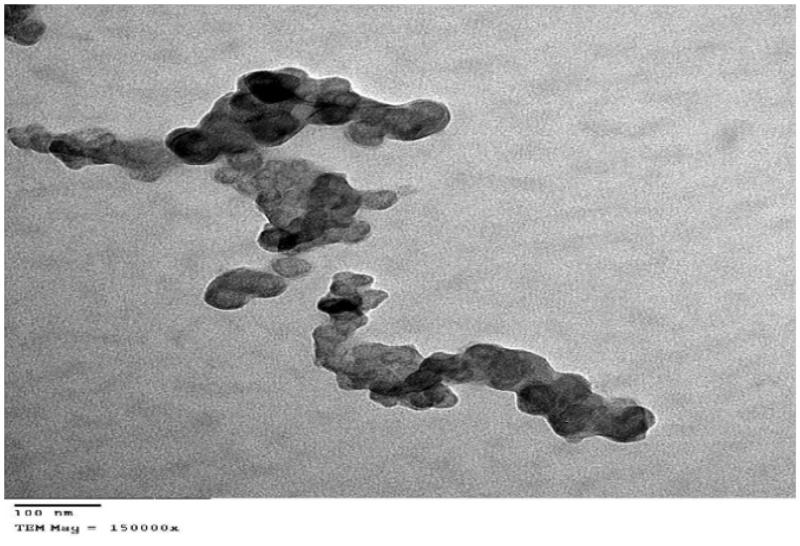
Transmission electron micrographs of Zal-loaded SNEDDS (F12) with magnification of 150 kx.

Transmission electron micrographs showed that the optimized Zal-SNEDDS (F12) is matched with the results of particles size measurement as they are nano-sized (Figure 4). Micrographs also revealed the existence of SNEDDS droplets in a spherical shape, and they appear as dark globules.

### *In-vitro* dissolution studies of CF12 (zal-loaded SNEDDS capsules)

**Figure 5.**
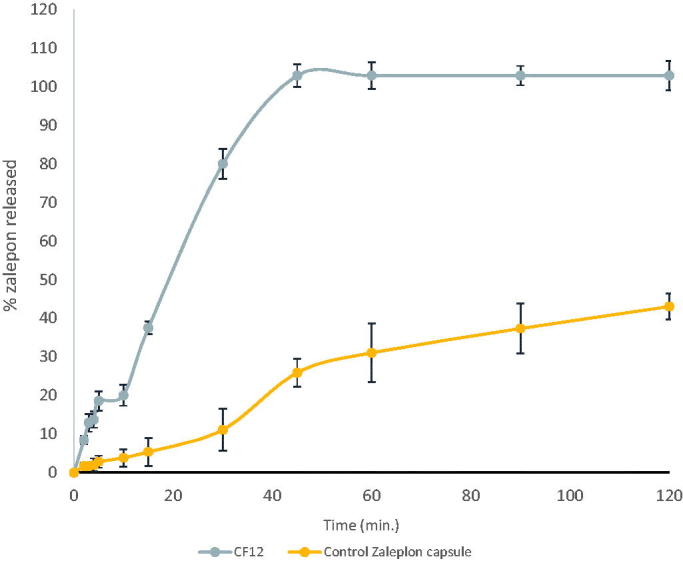
In-vitro dissolution profile of CF12 (Zal-loaded SNEDDS capsules).

Figure 5 shows the dissolution behavior of CF12 compared to zaleplon powder capsules. It was found that zaleplon powder released about 25.88 ± 3.6 after 45 minutes in HCl (pH 1.2) because of its hydrophobic nature (Janga et al., 2013), while a complete drug release was obtained from the optimized formula in the same medium (Syukri et al., 2018). Dissolution efficiency at 45 min was calculated for both optimized formulation and Zal powder capsule. DE%45 was 100 for the optimized formula while DE%45 for Zal powder capsule was 14.42. The increase in dissolution rate may be attributed to the enhanced zaleplon solubility. The surfactant incorporated in the formulation leads to high surface area and consequently small droplet size of the nano-emulsion generated (Zhang et al., 2008). Gupta et al., 2013 have discussed the relationship between the droplet size and the concentration of the surfactant being used and concluded that increasing the surfactant concentration could lead to droplets with smaller mean droplet size (Gupta et al., 2013).

Labrasol^®^ has an HLB value of 14 with hydrophilic properties which resulted in improved nano-emulsification (Hunter et al., 2012). The higher surfactant content, the better self-emulsification formation. On the other hand, transcutol (diethylene glycol monoethyl ether) as a cosurfactant was used to improve drug loading and time required for self-nano emulsification (Date et al., 2010). Transcutol lowers the interfacial tension to a very small value, decreases the bending stress of interface, and helps the formation of interfacial film with flexibility. This flexibility facilitates different curvatures required to form microemulsion from its component (Kawakami et al., 2002; Gupta et al., 2013). Therefore, the addition of cosurfactant improves drug release.

It was observed that the dissolution of the optimized formula (20% Labrafil and 1:1 Labrosol:Transcutol) in HCl exhibited Non-Fickian transport according to Korsmeyer-Peppa's release model which means anomalous transport and the release is controlled by a combination of diffusion and polymer relaxation, and also followed diffusion kinetics. Ahmed et al.,^2014^ showed that the Kinetic treatment results of the prepared glimepiride (GMD)-loaded self nano emulsifying delivery systems patches revealed also non-Fickian (anomalous) transport (Ahmed et al., 2014).

### Clinical pharmacokinetic parameters after oral administration of Zal-SNEDDS

**Figure 6.**
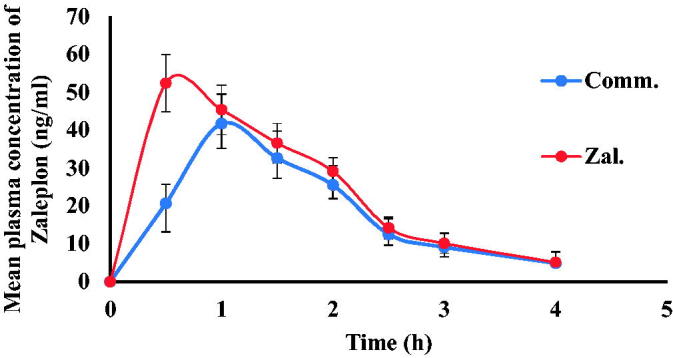
Mean plasma concentration of Zal-SNEDDS and commercial product.

The peak concentration (*C*_max_) and its time (*T*_max_) were obtained directly from the serum concentration versus time profile. The area under the curve (*AUC*_0→t_) was calculated by using the trapezoidal rule method. Pharmacokinetic study was conducted to assess Zal- SNEDDS oral bioavailability. The mean serum concentration versus time profiles of zaleplon following oral administration of Zal-SNEDDS formulation in comparison to commercial product is shown in Figure 6. It was found that *C*_max_ following Zal-SNEDDS administration (52.37 ± 2.66) was significantly higher compared to the commercial product (41.71 ± 2.44) at (*p* < .023). Moreover, the time to reach the peak concentration (*T*_max_) following Zal-SNEDDS administration (0.506 ± 0.028) was extremely reduced compared to *T*_max_ following commercial product administration (1.027 ± 0.048) at (*p* < .0003). However, the half-life (*t_1/2_*) remained non-significant between them. It was 1.1 hr and 1.02 hr for Zal-SNEDDS and commercial product, respectively. The area under the concentration-time curve (AUC) values which indicate the extent of absorption was 102 ± 1.77 ng h/mL following Zal- SNEDDS administration and was significantly higher compared to commercial product (80 ± 3.33 ng h/mL) (*p* < .002).

Overall, it is apparent from the results that the rate and extent of absorption of zaleplon have been markedly improved from Zal-SNEDDS compared to the commercial product.

## Sleep quality assessment among psychiatric patients treated with Zal-SNEDDS

The Pittsburgh Sleep Quality Index (PSQI) is a practical, brief and widely spread self-reported questionnaire for measuring the quality of sleep (Buysse et al., 1989). PSQI gives a single score representing overall sleep quality, which integrates qualitative and quantitative aspects of sleep; scores above 5 indicate a potential sleep problem. The PSQI can identify the nature and possible causes of sleep problems to help direct management, as well as subscale scores which indicate the type of sleep problems (sleep duration, latency, disturbances, quality, efficiency, daytime dysfunction, and the use of sleep medication) (Faulkner & Sidey-Gibbons, 2019).

In this study the total sample of the patients was on the average and predominantly females, the majority of patients were diagnosed as a mood disorder, and they were kept on a mood stabilizer and antidepressant. At the beginning of the research, the two groups were comparable regarding sex, marital status, education, occupation, mental illness and medication with no significant difference in age (Table 5).

**Table 5.** Descriptive data of the patients.

	Zal-loaded SNEDDS (*n* = 20)	Commercial product (*n* = 20)
**Age (years)**		
Median (Min–Max.)	36 (24–56)	36 (22–53)
Mean ± SD.	39.6 ± 2.34	37.05 ± 2.16
**Gender**		
Male	8 (40%)	8 (40%)
Female	12 (60%)	12 (60%)
**Marital status**		
Divorced	2 (10%)	2 (10%)
Married	11 (55%)	11 (55%)
Single	6 (30%)	6 (30%)
Widow	1 (5%)	1 (5%)
**Education**		
<6 years	9 (45%)	10 (50%)
6–12 years	9 (45%)	6 (30%)
>12 years	2 (10%)	4 (20%)
**Occupation**		
Manual	7 (35%)	10 (50%)
Jobless	8 (40%)	4 (20%)
Professional	5 (25%)	6 (30%)
**Mental Illness**		
BAD	7 (35%)	6 (30%)
GAD	4 (20%)	4 (20%)
MDD	7 (35%)	8 (40%)
SCZ	2 (10%)	2 (10%)
**Medications**		
SSRIs	12 (60%)	13 (65%)
Antipsychotics	1 (5%)	1 (5%)
Mood Stabilizers	7 (35%)	6 (30%)

BAD: Bipolar Affective Disorder; GAD: Generalized Anxiety Disorder; MDD: Major Depressive Disorder; Scz: Schizophrenia; SSRIs: Selective Serotonin Reuptake Inhibitors.

Baseline PSQI was done before giving any medications and revealed a high prevalence of sleep disturbances in patients with different psychiatric disorders. Total PSQI was (13.1 ± 0.463) in the first group and (12.85 ± 0.318) in the second group which indicated a very poor sleep quality. However, there was no statistical significance. The analysis of each component also reported complex and multiple problems in both groups that exhibit poor subjective sleep quality, increased sleep latency, poor sleep efficacy, prolonged sleep duration, and daytime dysfunction with no statistical significance between both groups (Table 6).

**Table 6.** Baseline PSQI in the studied groups.

	Zal-SNEDDS (*n* = 20)	Commercial product (*n* = 20)	*p* value
**Subjective sleep quality**			
Median (Min – Max.)	3 (1–3)	2 (2–3)	
Mean ± SD.	2.65 ± 0.131	2.45 ± 0.114	.168
**Sleep latency**			
Median (Min – Max.)	2 (1–3)	2 (1–3)	.844
Mean ± SD.	2.05 ± 0.153	2.1 ± 0.1	
**Sleep duration**			
Median (Min – Max.)	2 (0–3)	2 (0–2)	.916
Mean ± SD.	1.5 ± 0.212	1.5 ± 0.154	
**Sleep efficiency**			
Median (Min – Max.)	2 (1–3)	2 (1–3)	.797
Mean ± SD.	1.75 ± 0.143	1.7 ± 0.147	
**Sleep disturbance**			
Median (Min – Max.)	2 (1–3)	2 (1–3)	.661
Mean ± SD.	1.7 ± 0.147	1.6 ± 0.134	
**Use of sleep medication**			
Median (Min – Max.)	1 (0–2)	1 (0–2)	1
Mean ± SD.	0.95 ± 0.153	0.95 ± 0.153	
**Daytime dysfunction**			
Median (Min – Max.)	2.5 (2–3)	3 (2–3)	
Mean ± SD.	2.5 ± 0.115	2.55 ± 0.114	.766
**Total PSQIs**			
Median (Min – Max.)	13 (10–17)	13 (10–15)	
Mean ± SD.	13.1 ± 0.464	12.85 ± 0.319	.869

Total PSQI: Total Pittsburgh Sleep Quality Index.

Results of the Mann- Whitney rank sum test showed that the comparison between the before-treatment data of commercial and Zal- SNEDDS groups in all the seven aspects of the sleep quality in addition to the total index were found non-significant *p* value >.05 (Table 6). Results indicate that both groups were similar in the aspects of sleep quality which may support the results of the after-treatment data comparison between the two groups.

**Figure 7.**
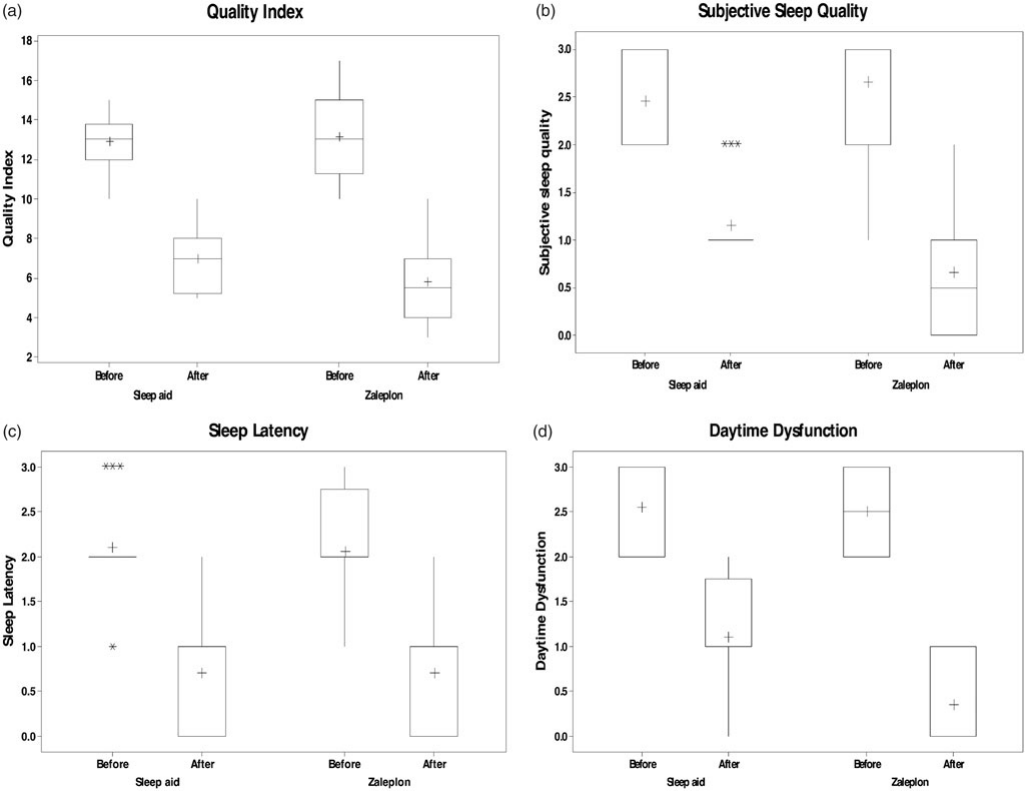
PSQI in Zal- SNEDDS group before and after treatment.

The total PSQI mean scores were higher in both groups (13.1 ± 0.463 in first group and 12.85 ± 0.318 in second group), indicating very poor sleep quality and this is in agreement with a study done by Doi et al. (2000) and Müller et al. (2016). Participant PSQI subscale scores were also similar to those from research using this instrument in a similar sample (Hedner et al., 2000; Müller et al., 2016), where sleep quality, sleep latency, and daytime dysfunction received higher scores on the PSQI. Patients administration of sleep medication scored very low, which should suggest minimal problems, and this is in agreement with Doi et al 2000 (Faulkner & Sidey-Gibbons, 2019). Treatment with both Zal- SNEDDS and commercial product significantly improved the total score of PSQIs (5.8 ± 0.451 in the first group and 6.95 ± 0.373 in the second group) with a statistical significance (*p* = .042) (Figure 7(a)). The analysis of each component of PSQIs revealed that treatment with both drugs was associated with higher subjective sleep quality (Figure 7(b)), reduced sleep latency (*p* = .009) (Figure 7(c)), and improvement in daytime function (Figure 7(d)) (*p* < .001) and (Table 7).

**Table 7.** Comparison of PSQI between the studied groups after treatment.

	Zal-loaded SNEDDS (*n* = 20)	Commercial product (*n* = 20)	*p* value
**Subjective sleep quality**			
Median (Min – Max.)	0.5 (0–2)	1 (1–2)	
Mean ± SD.	0.65 ± 0.167	1.15 ± 0.0819	.009**
**Sleep latency**			
Median (Min – Max.)	1 (0–2)	1 (0–2)	1
Mean ± SD.	0.7 ± 0.128	0.7 ± 0.128	
**Sleep duration**			
Median (Min – Max.)	1.5 (0–2)	1 (0–2)	.691
Mean ± SD.	1.3 ± 0.179	1.25 ± 0.143	
**Sleep efficiency**			
Median (Min – Max.)	2 (1–3)	1 (1–2)	.25
Mean ± SD.	1.65 ± 0.15	1.4 ± 0.112	
**Sleep disturbance**			
Median (Min – Max.)	0 (0–2)	1 (0–2)	.847
Mean ± SD.	0.65 ± 0.182	0.65 ± 0.15	
**Use of sleep medication**			
Median (Min – Max.)	0 (0–2)	1 (0–2)	.486
Mean ± SD.	0.5 ± 0.136	0.65 ± 0.15	
**Daytime dysfunction**			
Median (Min – Max.)	0 (0–1)	1 (0–2)	
Mean ± SD.	0.35 ± 0.109	1.1 ± 0.143	<.001**
Total PSQIs*			
Median (Min – Max.)	5.5 (3–10)	7 (5–10)	
Mean ± SD.	5.8 ± 0.451	6.95 ± 0.373	.042*

Results of the Mann- Whitney rank sum test showed that the comparison between the after-treatment data of commercial product and Zal-SNEDDS groups showed that Zal-SNEDDS results have an advantage over commercial product in two of the seven aspects (subjective sleep quality and daytime dysfunction) with *p* value <.01. On the other side, no significant results were found in the comparison between the other five aspects of sleep quality indicators with *p* value >.05. The total index has also shown significant results for the favor of the Zal- SNEDDS over the commercial product and most likely due to the significant differences found in sleep quality and daytime dysfunction with *p* value <.05 (Table 8).

**Table 8.** PSQI in Zal- SNEDDS group before and after treatment.

	Median	Q_1_	Q_3_	*p* value
**Subjective sleep quality**				<.001**
Before treatment	3	2	3	
After treatment	0.5	0	1	
**Sleep latency**				
Before treatment	2	2	2.75	<.001**
After treatment	1	0	1	
**Sleep duration**				
Before treatment	2	1	2	.375
After treatment	1.5	1	2	
**Sleep efficiency**				
Before treatment	2	1	2	.547
After treatment	2	1	2	
**Sleep disturbance**				
Before treatment	2	1	2	<.001**
After treatment	0	0	1	
**Use of sleep medication**				
Before treatment	1	0.25	1	.027*
After treatment	0	0	1	
**Daytime dysfunction**				
Before treatment	2.5	2	3	
After treatment	0	0	1	<.001**
**Total PSQIs**				
Before treatment	13	11.25	15	
After treatment	5.5	4	7	<.001**

Total PSQI: Total Pittsburgh Sleep Quality Index.

The data also showed that zal- SNEDDS formula reduces sleep latency (*p* < .001) and reduce the use of sleep medication (*p* = .027) (Table 8).

Results of the Wilcoxon Signed Rank Test showed that five aspects out of the seven sleep quality aspects in addition to the total index have been improved significantly as the results showed significant differences at *p*-value of <.001 between the before and after scores under the treatment with Zal-SNEDDS. Sleep duration and efficiency have not improved on the side and differences were found non-significant at *p*-value >.05 (Table 8).

After treatment with Zal-SNEDDS, there is a significant reduction in total PSQIS (indicating good sleep) with improved subjective sleep quality, reduced sleep latency, improved daytime function and reduced sleep disturbance and this is in agreement with Hedner et al. (2000), Sabbatini et al. (2004) and Huang et al. (2011).

It was found that patients receiving Zal-SNEDDS exhibited statistically significant improvement in subjective sleep quality and reduction in sleep latency compared to those who received commercial product. However, according to our knowledge no available study that compares the effect of SNEDDS and commercial zaleplon formula.

## Conclusion

SNEDDS have improved Zal solubility and enhanced its oral bioavailability. This can help reducing its dose and therefore avoid hallucinations related to high doses. Clinically, Zal-SNEDDS have ehnanced various sleep quality parameters including improved subjective sleep quality, reduced sleep latency, improved daytime function and reduced sleep disturbance among psychiatric patients. Therefore, it may be considered a good choice to enhance the quality of sleep.
